# A Rare Case of Adult Vocal Cord Hemangioma: A Case Report and Literature Review

**DOI:** 10.7759/cureus.44042

**Published:** 2023-08-24

**Authors:** Hiroshi Okuda, Mitsuhiro Aoki, Natsuko Ueda, Takenori Ogawa, Hideki Mori

**Affiliations:** 1 Otolaryngology - Head and Neck Surgery, Gifu University Graduate School of Medicine, Gifu, JPN; 2 Otolaryngology, Ogaki Tokushukai Hospital, Ogaki, JPN; 3 Otolaryngology, Gifu University Graduate School of Medicine, Gifu, JPN; 4 Pathology, Ogaki Tokushukai Hospital, Ogaki, JPN

**Keywords:** vocal cord polyp, laryngeal microsurgery, narrow band imaging, adult, vocal cord hemangioma

## Abstract

Infantile laryngeal hemangiomas are relatively common. However, adult vocal cord hemangiomas are extremely rare. A 46-year-old woman was referred to our department for hoarseness, which continued for 18 months. A laryngeal fiberscope revealed a small protuberant tumor resembling a polyp on her right vocal cord, and the narrow-band imaging showed abundant vascularity. Laryngeal microsurgery with a cold instrument under general anesthesia completely resected the tumor on the vocal cord. Histopathologically, the resected tumor consisted of vessels with thick walls and was diagnosed as a cavernous hemangioma of the vocal cord. After the surgery, she has never complained of hoarseness and has had no local recurrence for six months.

## Introduction

Laryngeal hemangiomas are endothelial vascular tumors categorized into infantile and adult hemangiomas. Infantile laryngeal hemangiomas are common, with an incidence rate of 0.8%-5% in infants [1,2]. The tumor develops between three and seven weeks after birth, proliferates for an average of three to five months, and then involutes over several years [3]. In children, it is typically located at the subglottic level [4]. Symptoms such as stridor, barking cough, and feeding problems may occur as the tumor grows, and there is a fatal risk of airway obstruction. Surgical resection with microlaryngoscopy using laser ablation has been selected as a treatment strategy for infantile laryngeal hemangiomas, while the administration of propranolol has been reported to decrease the cutaneous and airway hemangioma size drastically [4,5].

On the other hand, the incidence of laryngeal hemangioma in adults is unknown due to the rarity of reports. It can be located in different areas than in children, such as the supraglottic region, followed by the glottic and subglottic regions [6]. Symptoms vary depending on the primary site and include dysphagia and hoarseness. Unlike in children, there is no evidence of the utility of propranolol in adults, thus surgery is the preferred treatment [4,7].

Here, we report an adult case of vocal cord hemangioma, which was completely resected with microscopic surgery.

## Case presentation

**Figure 1:**
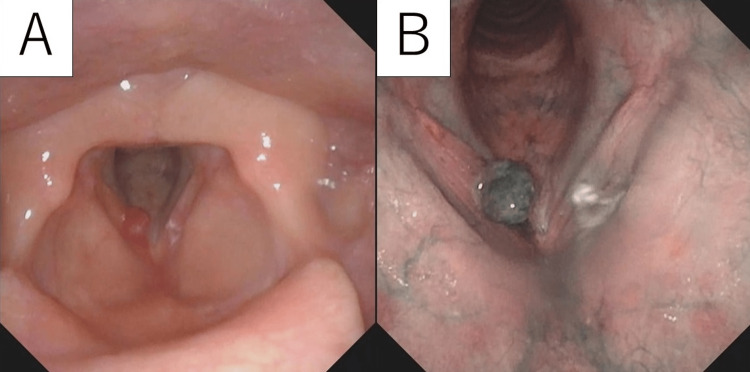
Laryngoscopy images of the tumor. (A) White light (WL). (B) Narrow-band imaging (NBI). The tumor was located on the anterior third of the middle of the right vocal cord. The surface was smooth and globular, showing lip-deep red in WB and dark green in NBI.

A 46-year-old female nursery teacher presented to our department with a chief complaint of hoarseness that continued for 18 months. She did not have any history of previous illness, drinking, or smoking. The laryngeal fiberscope revealed a mass on the anterior third of the right vocal cord, slightly close to the anterior commissure. The pale red-colored tumor had a nodular shape with a smooth surface. There were no abnormal characteristics for neovascularization, but abundant vascularity was seen on narrow-band imaging (NBI) (Figures 1A, 1B). The contralateral vocal cord displayed no abnormalities. Furthermore, the mobility of both vocal cords was normal.

As NBI findings were different from those of usual vocal cord polyps, a hemangioma was suspected. Surgical excision was planned without outpatient biopsy. During the laryngeal microsurgery, microscopic observation revealed that the tumor was pedunculated and was attached to the edge of the vocal cord epithelium. The tumor was gently pulled and excised by micro-scissors. Although persistent bleeding occurred at the time of excision, it was controlled using cotton balls containing adrenaline.

**Figure 2:**
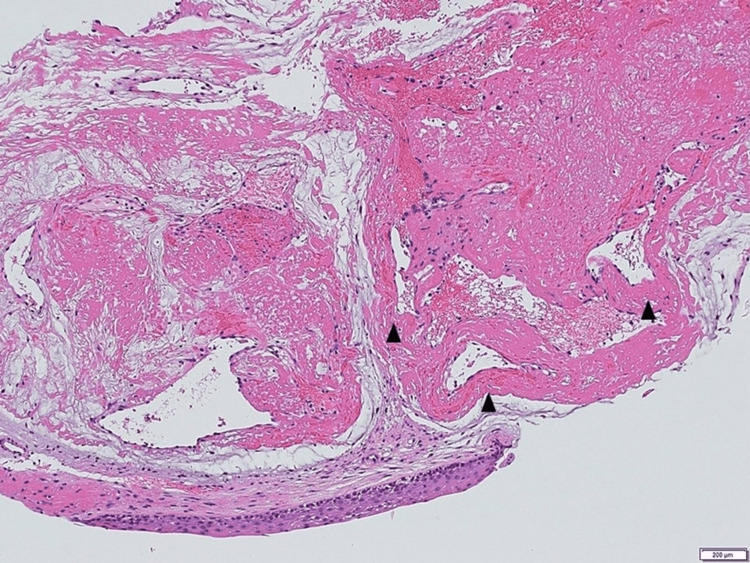
Histological findings of the tumor. Variably sized blood vessels with slightly thicker walls showing hemangioma. The surface epithelium of the vocal cord disappeared with bleeding (hematoxylin and eosin; original magnifications 100×).

Histopathological findings revealed that the tumor was composed of vessels with thick walls, and the surface epithelium of the vocal cord disappeared with bleeding. The tumor was diagnosed as a cavernous hemangioma of the vocal cord (Figure 2).

**Figure 3:**
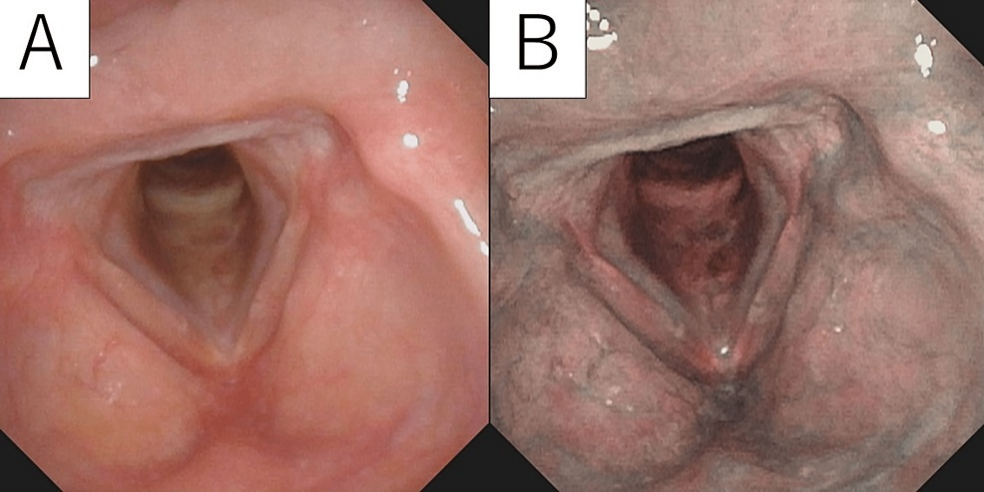
Laryngoscopy images of the tumor after surgery. (A) White light (WL). (B) Narrow-band imaging (NBI). The nodular tumor lesions on the right vocal cord disappeared, and the remaining mucosal tissue was smooth.

Follow-up laryngeal fiberscope performed on an outpatient basis showed no tumor recurrence, and normal vocal cord mucosa was preserved (Figures 3A, 3B). She has never complained of hoarseness after the surgery.

## Discussion

Vascular tumors of the head and neck in adults commonly arise in the cheek, tongue, lip, and scalp and rarely in the maxillary sinus and external ear [8,9]. However, vascular lesions in the larynx in adults are very rare, with most cases occurring in the supraglottic area. Hemangiomas of the vocal cord are extremely rare, and only a few case series and case reports have been published [10-14]. Of the 12 patients for whom detailed information was available in these articles, the average age was 49.5 years, with nine males and three females. The tumor bases were anterior in nine cases, posterior in one case, and anterior commissure in two cases. Excision with laryngeal carbon dioxide laser was selected in four cases, and cold steel was selected in five cases (information was not available in three cases). Regarding the choice of the resection method, there was no obvious tendency for the size of the tumor and the width of the base. Histological types were cavernous hemangioma in nine cases and capillary hemangioma in three cases.

Histologically, hemangiomas are classified into capillary, cavernous, and mixed types. Cavernous hemangiomas are more common and characterized by larger, thin-walled vascular lumens than capillary hemangiomas, which are mixed with blood vessels in varying proportions. The proliferation of the adipose tissue or smooth muscle bundles may be seen.

The onset factors are unknown; however, male sex, smoking, trauma such as intubation, and voice overuse are considered risk factors [15]. Our patient worked as a nursery teacher, and voice overuse may have contributed to hemangioma development.

The internal qualitative evaluation and blood flow of the tumor evaluated through echography and contrast-enhanced magnetic resonance imaging are helpful in diagnosis. However, these evaluations might be difficult because of the small tumors, as seen in our case. Biopsies are not usually taken in hemangiomas.

NBI allows the in-depth visualization of vessel patterns of the tumor surface by illuminating with the light of two narrow bands (blue light, 390-445 nm; green light, 530-550 nm) that are absorbed by hemoglobin [16]. NBI enables clear visualization of intrapapillary capillary loops (IPCLs) which are microvascular and arise perpendicularly from branching vessels in squamous subepithelium. In normal squamous epithelium, IPCLs are not conspicuous.

On the contrary, reports on the qualitative diagnosis of benign tumors using NBI are limited. The National Institute for Health and Care Excellence (NBI International Colorectal Endoscopic) classification is based on narrow-band images of colon polyps [17]. The classification uses staining, vascular patterns, and surface patterns to distinguish between hyperplastic and adenomatous colon polyps. According to Ogasawara et al., NBI displays the tumor surface in a cavernous hemangioma in the sigmoid colon as green [18]. The findings were similar to our case, where an enlarged image of the lumen of the capillary vessel, the histological characteristic of cavernous hemangiomas, was visualized by NBI. Although this is not specific to hemangiomas because this finding is also observed in inflammatory mucosal lesions, it may be useful for evaluating the amount of blood flow. Therefore, NBI is helpful for benign tumors as a tool for judging vascularity.

## Conclusions

Surgical resection, cryosurgery, radiation therapy, and laser surgery have been regarded as the treatment options for laryngeal hemangiomas. Our patient was treated with microscopic surgery using micro-scissors, resulting in complete excision with minimal scarring to the vocal fold.
